# Misleading about MBT in Oslo

**DOI:** 10.1186/s12888-019-2193-5

**Published:** 2019-07-04

**Authors:** Sigmund Karterud, Elfrida Hartveit Kvarstein

**Affiliations:** 1 Institute for Mentalizing, Svartediksveien 6a, 5009 Bergen, Norway; 2 Oslo University Hospital, Kirkeveien 144, Oslo, Norway

**Keywords:** Mentalization-based treatment, Borderline personality disorder, Correction

## Abstract

In this correspondence we correct some misleading information about mentalization-based treatment in Oslo, Norway.

## Main text

We are very much in sympathy with the therapeutic project, mentalization-based treatment (MBT) for borderline patients, recently being reported by our Swedish colleagues Löf et al. [1] in BMC Psychiatry. However, we have to correct some misleading information that Löf and co-workers convey about one of our previous studies [2]. Our study concerned 64 borderline patients who had received MBT and who were compared to a representative sample of 281 borderline patients who had received psychodynamic treatment. Löf et al. [1] have some critical remarks to this study. They assert 1) that our sample was not “community-based” (p. 2), 2) that it was “not clear how BPD diagnosis was established, nor whether diagnoses were valid and reliable since no information was provided on possible exclusion criteria” (p. 2), and 3) that the effect size (EZ) of our study on “general psychiatric symptoms” (SCL-90R) was 1.05 (p. 7).

These assertions are incorrect. In our publication we 1) report that patients were referred to specialist outpatient treatment in Oslo, the sample thus being “community-based” (p. 3). In fact, the outpatient department had a regional responsibility for the city of Oslo. Based upon our original text, this might be a misunderstanding from Löf et al. It is harder to understand the reasons for the next points. 2) Under the heading “Diagnostic skills and reliability” we report that all patients were diagnosed with SCID-II interviews and we even report the reliability of the procedure (p. 4). 3) On p. 8 we report that the effect size of general psychiatric symptoms (brief version of SCL-90R) was 1.79 (not 1.05).

Towards the end of their article, Löf et al. [1] discuss their effect size (d = 0.58) on psychiatric symptoms. It seems unwarranted to compare it with ours (d = 1.79), since ours is an estimate at 3 years for a treatment program that lasted up to 3 years, while the Swedish program terminated after 18 months. As the authors write, psychiatric symptoms might decline even after termination of psychotherapy. However, a sounder way to increase treatment results, would be to prolong treatment time. This is not so costly if one invests in mentalization-based group psychotherapy (MBT-G).

## Data Availability

Not applicable.

## Abbreviations

BPD: Borderline personality disorder
EZ: Effect size
MBT: Mentalization-based treatment
MBT-G: Mentalization-based group psychotherapy
