# Supplementary material for: Misleading about MBT in Oslo
Source: BMC Psychiatry. 2019 Jul 4;19:208. doi: 10.1186/s12888-019-2193-5 (PMC6609381; doi:10.1186/s12888-019-2193-5)
Supplement: Related texts

# Reply: Authors’ response

**Authors:** David Clinton

Thank you for the opportunity of replying to the points raised by our discussion of Kvarstein et al (2015) [2].

Prof. Karterud is right that we failed to report the correct effect size from their study in relation to changes in the BSI-18, which should be *d*=1.79 instead of *d*=1.05 for MBT patients. We sincerely apologise for this mistake. However, we take issue with their other contentions that we present “incorrect” information about their study. Our discussion reflects the fact that the paper by Kvarstein and colleagues fails to provide sufficient clarity about their methods and results. Accordingly, we believe that a reasonable scientific approach should be to interpret the quality of their work conservatively.

As regards the question of the community-based nature of their sample, Prof. Karterud writes that the outpatient department to which patients were referred “had a regional responsibility for the city of Oslo”. This fact is, however, missing from their report. In the paper the authors say little about the unit where patients were treated and it’s relation to the health service. They do not elaborate on the selection process, how it was carried out, what sort of referral system was used, and whether the selection system remained the same during the long period that the study covers. Such information is essential for judging whether their study comprised a representative community-based sample.

Neither is it clear, as we point out, how BPD diagnoses were made, nor whether diagnoses were valid and reliable. It is true that the authors used SCID-II interviews at baseline, and reported kappa for BPD. However, they use an extremely small sub-sample comprising 24 of 345 patients for estimating reliability. We have no way of knowing how representative these 24 patients were, how well the remaining 321 cases were diagnosed, or whether specific exclusion criteria were applied to the sample as a whole or during particular periods. All the authors report is that a few patients were excluded due to participation in an RCT, while others were excluded during a transitional period. The study covers patients treated over a 20-year period, but there is no information about how the quality of BPD diagnoses and psychiatric assessment might have varied during this period, raising further questions about the reliability and validity of diagnoses.

Prof. Karterud raises questions about comparing effect sizes in our two studies. Although comparisons are made more difficult by the differing lengths of treatment in the studies, as Prof. Karterud points out, conclusions regarding effect sizes in Kvarstein et al are hampered by the lack of clarity about the number of persons their pre-post comparisons are based on. A strength of our study is that our flowchart contributes to a high degree of transparency. Kvarstein et al report percentages under “repeated outcome assessments”, but it is not known who is being compared, and whether the high effect size they report could be due to the authors following up the healthiest patients (i.e. possible selection bias).

In sum, although we did regrettably make a mistake in our reporting of effect size from Kvarstein et al, we believe that the authors make important methodological omissions in the reporting of their data that allow for questions to be raised about the community-based nature of their sample, the validity and reliability of BPD diagnoses, and the relevance of their effect sizes.
